# Determination of Aflatoxin B_1_ in Feedstuffs without Clean-Up Step by High-Performance Liquid Chromatography

**DOI:** 10.1155/2018/4650764

**Published:** 2018-06-24

**Authors:** Sasiprapa Choochuay, Jutamas Phakam, Prakorn Jala, Thanapoom Maneeboon, Natthasit Tansakul

**Affiliations:** ^1^Department of Veterinary Pharmacology and Toxicology, Faculty of Veterinary Medicine, Kasetsart University, 10900, Bangkok, Thailand; ^2^Center for Advanced Studies for Agriculture and Food, KU Institute for Advanced Studies, Kasetsart University (CASAF, NRU-KU), 10900, Bangkok, Thailand; ^3^Faculty of Veterinary Medicine, Kamphaengsaen Campus, 73140, Nakhon Pathom, Thailand; ^4^Kasetsart University Research and Development Institute, 10900, Bangkok, Thailand

## Abstract

A reliable and rapid method has been developed for the determination of aflatoxin B_1_ (AFB_1_) in four kinds of feedstuffs comprising broken rice, peanuts, corn, and fishmeal. A sample preparation was carried out based on the QuEChERS method with the exclusion of the clean-up step. In this study, AFB_1_ was extracted using acetonitrile/methanol (40/60 v/v), followed by partitioning with sodium chloride and magnesium sulfate. High-performance liquid chromatography with precolumn derivatization and fluorescence detection was performed. The coefficients of determination were greater than 0.9800. Throughout the developed method, the recovery of all feedstuffs achieved a range of 82.50-109.85% with relative standard deviation lower than 11% for all analytes at a concentration of 20-100 ng/g. The limit of detection (LOD) ranged from 0.2 to 1.2 ng/g and limit of quantitation (LOQ) ranged from 0.3 to 1.5 ng/g. The validated method was successfully applied to a total of 120 samples. The occurrence of AFB_1_ contamination was found at the following concentrations: in broken rice (0.44-2.33ng/g), peanut (3.97-106.26ng/g), corn (0.88-50.29 ng/g), and fishmeal (1.06-10.35 ng/g). These results indicate that the proposed method may be useful for regularly monitoring AFB_1_ contamination in feedstuffs.

## 1. Introduction

Aflatoxins (AFs) are secondary metabolites of fungi (e.g.,* Aspergillus flavus* and* A. parasiticus*). AFs occur naturally and can be found in common food and feedstuffs such as rice, peanuts, corn, fishmeal, and soybean meal [1–3]. The four major analogues—aflatoxins B_1_ (AFB_1_), B_2_ (AFB_2_), G_1_ (AFG_1_), and G_2_ (AFG_2_)—are the most important members because they all pose a potential risk to human and animal health if food and feedstuffs have been contaminated. In particular, the toxicity of AFB_1_ can range from levels that may cause immune system suppression to the induction of teratogenic, mutagenic, and carcinogenic activities [4, 5], which is collectively classified as a carcinogen (Group 1) by the International Agency for Research on Cancer (IARC) [6]. In addition, feedstuffs comprise the first link of the food chain; therefore, there is a risk of AFB_1_ carryover from feedstuffs into animal tissues and/or biological fluids such as meat, milk, and eggs, which may eventually be hazardous for human consumption [7–9]. As a result of such adverse health effects of the toxin, it is necessary to have a sensitive, reliable, and accurate method for monitoring AFB_1_ level in feedstuffs.

High-performance liquid chromatography with fluorescence detection (HPLC-FLD) is considered as the most reliable instrument for the quantification of AFs due to its accuracy and high sensitivity. However, it requires clean-up steps involving immune affinity columns (IAC) to remove interferences and preconcentration of AFB_1_. IAC is also quite expensive, difficult to use, and time-consuming [10]. In this regard, a QuEChERS (Quick, Easy, Cheap, Effective, Rugged, and Safe) method, which was originally developed for pesticides and veterinary drug residue analysis, has been applied for the measurement of mycotoxins in a variety of matrices [10–12]. Commonly, it involves two steps: the first step is the extraction step based on a partitioning phase, and the second step is the dispersive-solid phase extraction step (d-SPE) using a combination of magnesium sulfate with different sorbents. The advantages of this method are that it is simple and reduces time consumption [13, 14].

In order to reduce sample handling and increase sample output efficiency, the aim of this study is focused on the optimization of sample preparation using QuEChERS-based extraction and optimizing the chromatographic conditions of an HPLC-FLD method with pre-column derivatization for quantification of AFB_1_ in the feedstuffs as well as a cross-sectional investigation of the contamination of AFB_1_ content in the feedstuffs using a QuEChERS-based method.

## 2. Materials and Methods

### 2.1. Chemicals and Reagents

AFB_1_ standard was purchased from Sigma-Aldrich (Madrid, Spain). Stock solution of AFB_1_ was prepared in methanol at a concentration of 10 mg/mL. From the stock standard solution, working solution was prepared by diluting AFB_1_ in methanol at a concentration of 1 mg/mL and stored at 4°C. Before use, the solution was brought to laboratory room temperature (25°C) and thoroughly shaken. Acetonitrile and methanol were of HPLC grade and supplied by ACILabscan (Thailand). Sodium chloride was obtained from Ajax Finechem Pty. Ltd. (New Zealand) and anhydrous magnesium sulfate was from Applicati Chem Panreac ITW Companies (Germany). Trifluoroacetic acid was purchased from ACROS Organics (Belgium) and water was purified (18 MΩ) on a Milli-Q Plus apparatus.

### 2.2. Optimization of the HPLC-FLD Analytical Method

To fulfill the purposes, several conditions, including a mobile phase, extraction solvents combined with the QuEChERS salt, volume of extraction, and precolumn derivatization solvents, were optimized to ensure an efficient determination of AFB_1_. Different combinations of the mobile phase solution, including methanol/water (50/50 v/v), acetonitrile/methanol/water (20/20/60 v/v/v), acetonitrile/methanol/water (16/22/62 v/v/v), and acetonitrile/methanol/water (15/15/70 v/v/v), were evaluated in order to optimize the retention time of the AFB_1_ and to enhance the sensitivity. In addition, the different mixtures of extract solvents that consisted of acetonitrile (100%), acetonitrile/methanol (40/60 v/v), and acetonitrile/water (50/50 v/v) were investigated. Rather than optimizing the ratio of extraction solvents alone, this study simultaneously evaluated the addition of different drying agents or salt; these included sodium chloride/magnesium sulfate (1/4 w/w) or sodium acetate/magnesium sulfate (1/4 w/w). The effect of extraction volume of the satisfied extraction solvent was tested by two different volumes of 10 and 20 mL. Moreover, the critical points of reconstitution of the precolumn derivatization with suitable solvent between 10% acetonitrile and the selected mobile phase were compared.

### 2.3. Optimization of Sample Preparation

The optimization of sample preparation was carried out using broken rice as a representative matrix. Briefly, the effects of different extraction solvents (acetonitrile 100%, acetonitrile/methanol 40/60 v/v, and acetonitrile/water 50/50 v/v) and salts (sodium chloride and sodium acetate) on the signal response of AFB_1_ were compared. After milling and homogenization, a 10 g sample was weighed into the extraction tube. Then, the sample was spiked by adding AFB_1_ standard solution and left at laboratory room temperature (25°C) for approximately 30 min to allow the solvent to evaporate. After leaving the samples to undergo a process of equilibration, the extraction solvents were added and shaken at 3000 g for 3 min to ensure that the solvents had mixed thoroughly with the entire sample. Thereafter, 1 g of the selected salt (sodium chloride) with 4 g of anhydrous magnesium sulfate was added to the mixture and the shaking procedure was repeated in order to induce phase separation and AFB_1_ partitioning. Subsequently, an aliquot of the supernatant layer (1 mL) was evaporated until dry under nitrogen gas. Following that, the precolumn derivatization of AFB_1_ was done in terms of how to enhance its sensitivity. The residue was reconstituted in 900 *μ*l of 10% acetonitrile or in the mobile phase that had been optimized in this study. After that, 100 *μ*l of trifluoroacetic acid was added, followed by an incubation period at 50°C for 15 min. The derivatized solution was then centrifuged at 1000 g for 5 min before HPLC-FLD analysis took place.

The influence of the volume of extraction solvent on the signal response of AFB_1_ was optimized (in this case, a mixture of acetonitrile/methanol 40/60 v/v was used as the extraction solvent). The volumes of extraction solvent compared were 10 mL and 20 mL. Subsequent extraction procedures were done as described above.

### 2.4. Validation of Methods

#### 2.4.1. Final HPLC Analysis Used in Validation Experiments

The apparatus used for the analysis was a reverse phase HPLC (Shimadzu, Kyoto, Japan) equipped with a fluorescence detector. The analytical column used was a Symmetry® C-18 3.9x150 mm with 5 *μ*m particle size from Waters (Massachusetts) and the guard column was Silfilter STD C-18 3.0x10 mm. The column was maintained at 40°C. Analysis was run at a flow rate of 1 ml/min by an isocratic mobile phase using a mixture of acetonitrile/methanol/water (15/15/70 v/v/v). The total run time was 20 min. An aliquot of a 10 *μ*l sample extract was injected into the chromatographic system and detection was carried out by a fluorescence detector (excitation and emission wavelengths were 360 and 440 nm, respectively). Chromatograms were displayed with Class VP LC software.

#### 2.4.2. Final Sample Preparation Method Used in Validation Experiments

10 g of the sample and 20 mL of acetonitrile/methanol (40/60 v/v) were added to an extraction tube. Extraction was achieved through shaking the sample at 3000 g for 3 min. Thereafter, phase partition was induced using 1 g of sodium chloride and 4 g of anhydrous magnesium sulfate with shaking at 3000 g for 3 min. Subsequently, 1 ml of the organic layer was evaporated until dry under nitrogen gas. After evaporation, a precolumn derivatization of AFB_1_ was prepared by reconstituting the residue in 900 *μ*l of 10% acetonitrile solution and adding 100 *μ*l of trifluoroacetic acid followed by incubation at 50°C for 15 min. Finally, the derivatized solution was centrifuged at 1000 g for 5 min and an aliquot was transferred to the vials for HPLC-FLD analysis which was described previously.

The method was validated according to SANCO/12571/2013 which demonstrates the conformity of the analytical performances with criteria established in regulation (EC) no. 178/2010 [15]. The guidelines recommend the validation procedure for linearity, specificity, limit of detection (LOD), limit of quantitation (LOQ), accuracy, and precision. This study used the maximum permitted levels (MPLs) for the legislated mycotoxin in animal feed under the Animal Feed Quality Control Act B.E. 2525 of Thailand at 100 ng/g to consider as a maximum fortified concentration for the samples.

Linearity was tested by external standardization using matrix calibration curves constructed from AFB_1_ standard solutions at 6 different concentrations within the range of 5-100 ng/g (5, 10, 20, 40, 60, and 100 ng/g). Analytical curves were established by plotting the peak areas which were used as the analytical signal response (y) versus the concentration of AFB_1_ (x).

The specificity of the method was evaluated by comparing the retention times in the blank sample matrices and the samples were spiked with 100 ng/g of AFB_1_ to ensure there was no interference in the retention time of the target analyte.

The sensitivity of the method was considered according to the LOD and LOQ. The LOD was calculated as the lowest concentration of the AFB_1_ giving a signal response 3 times greater than the average of the baseline noise obtained from 10 independent blank samples of each matrix (S/N 3:1) and the LOQ was defined as an AFB_1_ signal response 10 times greater than the average of the baseline noise obtained from 10 independent blank samples of each matrix (S/N 10:1).

The accuracy was tested through recovery studies by spiked AFB_1_ standard solution at 3 different concentration levels (equivalent to 20, 40, and 100 ng/g) into blank sample matrices. Six replicates of each concentration were prepared for each matrix. The level of precision, which is expressed as relative standard deviations (RSDs), was estimated by performing intraday repeatability, expressed as RSD_r_, and interday reproducibility, expressed as RSD_R_, by spiked blank sample with AFB_1_ standard solution at 3 concentration levels (20, 40, and 100 ng/g). Again, 6 replicates of each concentration were prepared for each matrix and determined within 1 day and in 3 consecutive days.

### 2.5. Limited Survey of AFB_1_ Contamination in Selected Feedstuffs

A total of 120 samples, including 30 samples of each kind of feedstuffs (broken rice, peanuts, corn, and fishmeal), were randomly collected from swine farms in the western region of Thailand. All samples were sealed in plastic bags and stored at -20°C until analysis.

## 3. Results and Discussion

### 3.1. Optimization of HPLC-FLD Conditions

To optimize the chromatographic separation, the study was carried out using four different solutions of mobile phase solvent as described above. The results showed that using methanol/water (50/50 v/v), acetonitrile/methanol/water (20/20/60 v/v/v), and acetonitrile/methanol/water (16/22/62 v/v/v) obtained poor signal responses and the AFB_1_ was not separated from the unretained compound. The best mobile phase which gave the highest signal response was obtained and that offered adequate chromatographic separation with acetonitrile/methanol/water (15/15/70 v/v/v). This might be due to the fact that a high polarity of the mobile phase combination was able to exclude nonpolar substances from the C-18 analytical column [16]. However, broadened peak may occur when the mobile phase is composed of water over 70% [10].

HPLC-FLD has been the most popular analytical instrument for AFB_1_ analysis. Nevertheless, fluorescent detection sensitivity of AFB_1_ requires a conversion to more highly fluorescent derivatives (AFB_2a_). Several derivatization methods are available, including precolumn treatment with trifluoroacetic acid or postcolumn derivatization with pyridinium hydrobromide [2, 17]. With an existence instrument, the method used the precolumn derivatization throughout the quantitative determinations. Generally, the residues were reconstituted using a mobile phase to reduce the solvent effects. However, in this study, the chromatographic response signal of the AFB_1_ derivative was unsatisfied when dissolved with mobile phase (acetonitrile/methanol/water 15/15/70 v/v/v). This might be due to the fact that methanol affects the trifluoroacetic acid derivatization and AFB_2a_ could potentially be degraded by methanol, which would then result in a poor signal response [18, 19].

### 3.2. Optimization of Sample Preparation

In quantifying the substance, the sample preparation has a crucial impact on the accuracy and precision of the results, especially when the sample was contaminated at very low level. Usually, the sample was extracted and partitioned using acidified acetonitrile and salt as a drying agent followed by the purification of extractant with d-SPE [11, 12]. To reduce sample handling, in this study, a simple extraction QuEChERS-based procedure without d-SPE clean-up step was applied.

Following the current protocol, the optimization method was carried out using 10 g of broken rice as a representative. The conventional extraction procedures of AFB_1_ from different matrices used a mixture of water and organic solvents such as acetonitrile, methanol, or acetone [20]. Therefore, different mixtures of extract solvents, which were acetonitrile (100%), acetonitrile/methanol (40/60 v/v), and methanol/water (50/50 v/v), were evaluated. An important characteristic of the QuEChERS procedure is the addition of salts, such as sodium chloride, sodium acetate, or sodium sulfate, which the typical QuEChERS method uses to separate interfering compounds from matrix and increase the solubility of the analyte into the organic phase [10, 14, 21]. Therefore, all of the extraction solvents were tested by adding either sodium chloride/magnesium sulfate (1/4 w/w) or sodium acetate/magnesium sulfate (1/4 w/w). The results clearly showed that the highest efficiency to extract AFB_1_ was achieved when using acetonitrile/methanol (40/60 v/v) combined with sodium chloride/magnesium sulfate (1/4 w/w). Generally, the initial QuEChERS method was developed for the determination of pesticide residue in fruits and vegetables which contain a high concentration of water (>80%), while feedstuffs have lower percentages of water composition than in those matrices. An earlier report suggested that the sample should be soaked in water prior to extraction [22]. However, the extraction procedure in this study was done without this soaking step.

Moreover, the present results showed that using acetonitrile (100%) and a mixture of acetonitrile/water (50/50 v/v) as an extraction solvent resulted in a low recovery of determination which is in accordance with Capriotti [23]. This might be due to the fact that AFB_1_ is normally soluble in moderately polar organic solvents, e.g., methanol or chloroform, and scarcely soluble in water. Consequently, AFB_1_ could not be completely extracted and remained in matrix [24]. Although acetonitrile/water (50/50 v/v) offered high signal response, its broadened tailing chromatogram overlapped with interference peaks. Moreover, the presence of a large amount of water in the solvent was adsorbed by matrices and tended to aggregate in the tube. Generally, the drying agents are anhydrous inorganic salts that acquire water for hydration. Therefore, the crystal form with large clumps of drying agent commonly occurred when exposed to water. Then, the results created a high viscosity of the extractant, reduced volume of supernatant layer, and extended duration for the evaporation step and a low signal response as well as percentage of recovery [10, 20].

The volume of extraction solvent (10 mL and 20 mL) was optimized. The results clearly showed that using 20 mL of extraction volume gave more satisfying data than using 10 mL. This is in agreement with an earlier study that used an adequate volume for extraction that increased the extraction efficiency by decreasing the matrix effect and enhanced the release of the AFB_1_ from the matrix resulting in high recoveries [10]. Therefore, 20 mL of acetonitrile/methanol (40/60 v/v) combined with sodium chloride (1g) and magnesium sulfate (4g) without any clean-up step was chosen as an extraction solution for AFB_1_ analysis.

### 3.3. Method Performance

**Figure 1:**
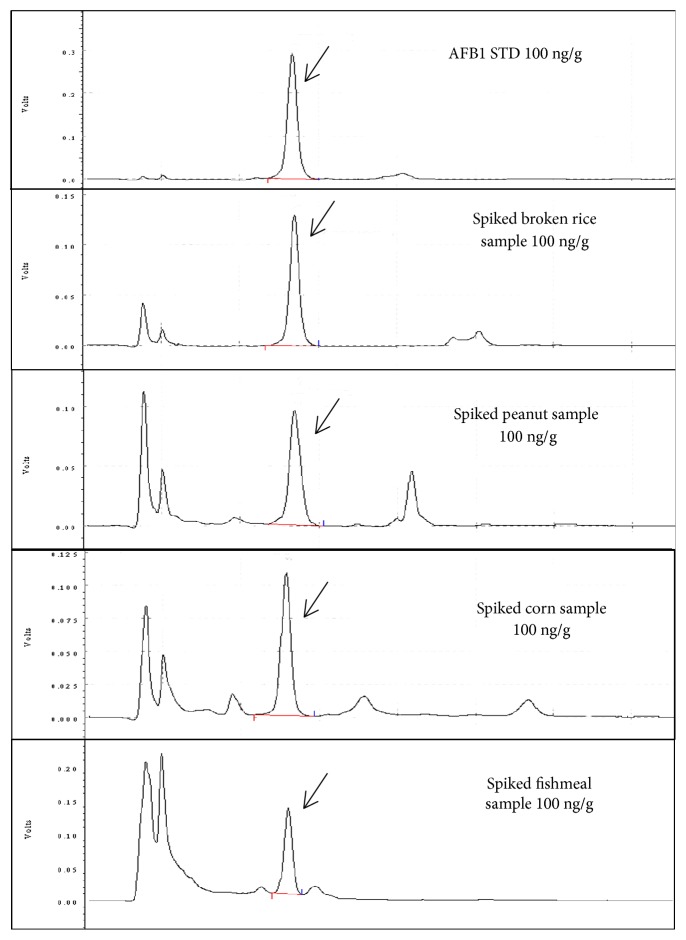
Chromatograms of AFB_1_ standard solution containing 100 ng/g and sample matrices (broken rice, peanut, corn, and fishmeal) were spiked with AFB_1_ standard solution at 100 ng/g.

Performance characteristics of the optimized method were established by a validation procedure with spiked feeding stuff samples to test linearity, specificity, accuracy, precision, LOD, and LOQ. As a result, the analytical curves showed good linearity within the working range (5-100 ng/g), with coefficients (R^2^) of determination higher than 0.9800. The discrimination between AFB_1_ and interference compounds was defined as specificity. The absence of any signal response close to the retention time of AFB_1_ in all matrices indicated that there were no matrix interferences in spite of the high complexity of the matrices. The obtained results have no coeluting peaks close to the retention time of the AFB_1_ (Figure 1), demonstrating that it was a specification method.

The sensitivity of the method was considered according to the LOD and LOQ (Table 1). As presented, the calculated values in all matrices were found to have the LOD between 0.2 and 1.2 ng/g. The LOQ was in the range of 0.3-1.5 ng/g. Both the LOD and LOQ values were satisfactory for the AFB_1_ detection in the feedstuffs, being far below the MPLs legislated values.

In order to check the accuracy of the method, recovery experiments were tested with a spiked AFB_1_ standard solution at 3 different concentration levels (20, 40, and 100 ng/g). Average recoveries for all feedstuffs were in the range of 82.50-109.85% (Table 1) which demonstrated the conformity of the guidelines for the recovery of analysis range of 70–110%, according to SANCO/12571/2013. RSDs were calculated under RSD_r_ and RSD_R_ conditions to evaluate the precision of the method. In the present study, the RSD_r_ and RSD_R_ of most samples were 1.02-6.77% and 0.57-6.58%, respectively, except for the fishmeal matrix which obtained RSD_r_ at 11% on the third day and RSD_R_ was found to be between 6.16 and 8.29% (Table 1). However, the performed method was in the acceptable range of RSDs of less than 20% with the performance criteria requirement of the EC [15]. Additionally, the obtained results comply well with the Thai regulations for the detection of AFB_1_ in feedstuff.

### 3.4. Occurrence of AFB1 from Collected Samples

The developed method was applied for the analysis of a total of 120 feedstuff samples including 30 samples of each type of feedstuffs of broken rice, peanuts, corn, and fishmeal which were randomly collected from swine farms in the western region of Thailand where there is a high density of livestock available. Several types of AFs analogues are naturally produced in food and feed. Obviously, AFB_1_ contamination in animal feed is the most predominant compared to other metabolites. Currently, of four major AFs analogues, AFB_1_ is the only metabolite with maximum permitted levels (MPLs) for animal feed set under Directive 2002/32. A guidance value of the AFs contamination by the European Food Safety Authority (EFSA) in animal feed is limited between 5 ng/g for compound feed of dairy and young animals and 20 ng/g for all other feed materials. In Thailand, the safe limits of AFB_1_ at 30-100 ng/g in animal feed (depending on the type of feed and animal species) have been regulated under the Animal Feed Quality Control Act B.E. 2525 of Thailand.

With regard to the data (Table 2), a low incidence (23.33%) with AFB_1_ trace level contamination in broken rice samples was determined by the validated method. It is possible that AFB_1_ is present mainly at the fungal invading area and remains on the outer layer of the grain which was removed by the degree of milling. As a result, AFB_1_ in broken rice is usually found at trace level [25–27].

However, postharvest factors, for instance, storage under inappropriate conditions and being exposed to rain, may lead to increased AFB_1_ contamination levels [28]. Likewise, Anjum [1] reported that AFB_1_ contamination in broken rice is affected by rain. In addition, Thieu [25] has reported that broken rice could be contaminated with AFB_1_ during harvesting and via a dehumidification process through sun-drying technique.

A high incidence of AFB_1_ contamination in peanuts (40%) with a wide range from 3.97 to 106.27 ng/g was found in this study. Among those, 11 samples were contaminated with an AFB_1_ level that exceeded the maximum levels set in Directive 2003/100/EC, amended Directive 2002/32 (20 ng/g), and 7 samples were contaminated with AFB_1_ level higher than the permissible limits as established by the Animal Feed Quality Control Act B.E. 2525 of Thailand (50 ng/g). In contrast, our current study showed that the contamination level was higher than that found in peanuts from Thailand in a study conducted by Thanida [29]. Moreover, 93-99% of the 200 peanut samples collected from 2012 to 2015 were found to have lower than 4 ng/g (unpublished in-house data). This may be due to underestimation detected by the ELISA method used in previous survey.

Corn (maize) is one of the major ingredients used in animal feed which susceptible to infection by mycotoxin-producing fungi and, consequently, has the potential to become contaminated with mycotoxins [30]. The current results showed that the incidence of AFB_1_ remained in 23 out of 30 corn samples (76.67%) with a wide range of concentrations (0.88-50.29 ng/g). Similarly, previous reports have shown that corn can become contaminated with AFB_1_ in various ranges. Recently, AFs in corn ingredient were found with a high prevalence [31]. Thanida [29] reported AFB_1_ contamination in corn samples from Thailand found ranging from 0.275 to 40 ng/g. As compared to the in-house data, 15% of the total 400 corn feed samples collected during 2012 to 2015 were contaminated with AFB_1_ ranging from 51 to 100 ng/g while 7% of the samples contained AFB_1_ over 100 ng/g (unpublished data). Our current study presented the notion that the AFB_1_ contents were within the safe limits under the Animal Feed Quality Control Act B.E. 2525 of Thailand (for feedstuff set as 100 ng/g) and the FDA guidance (100-300 ng/g).

Typically, AFB_1_ is not found in fishmeal because its matrix is unsuitable for the production of mycotoxin. It has been presented that AFB_1_ was not detected in any fishmeal samples [32]. However, fishmeal contaminated with AFB_1_ has been reported. Anjum [1] found AFB_1_24 ng/g from Pakistan during rainy season which was in accordance with the results of Mayahi [33] who found AFB_1_ contamination at an average of 15 ng/g. As detected in the present study, 20% of the sample was found to be contaminated with AFB_1_ in the range of 1.06-10.35 ng/g. This coincides with earlier results reported by Thanida [29] that AFB_1_ contamination in fishmeal was found in the range of 1.67-11.90 ng/g. This indicates that fishmeal as a feedstuff presents no risk of AFB_1_ contamination to animal health as the detected AFB_1_ was not above the MPLs.

## 4. Conclusion

A modified method based on the QuEChERS procedure by a single extraction step without employing a clean-up step was developed for AFB_1_ determination in feedstuffs. Good analytical results were obtained, including good linearity, specificity, accuracy, precision (repeatability and reproducibility), and analytical limits (LOD and LOQ). Therefore, it can be suggested as an alternative to expensive and time-consuming methods by using immune affinity columns or two steps of liquid/solid extraction procedure. Finally, an efficient and sensitive method was developed here that was applied to four different kinds of matrices which were detectable at low level. Considering that, the usefulness of the method could be applied as a regular monitoring method of AFB_1_ in broken rice, peanuts, corn, fishmeal, and their related matrices.

## Figures and Tables

**Table 1:** The results of validation method with mean value*∗*(accuracy and precision), n=6.

Matrices	Spiked level (ng/g)	%Recovery*∗*			Intra-day*∗* (%RSD_r_)			Inter-day*∗*(%RSD_R_)	LOD (ng/g)	LOQ (ng/g)
		Day 1	Day 2	Day 3	Day 1	Day 2	Day 3			
Broken rice	20	86.83	91.83	96.83	3.52	5.48	3.16	5.44	0.2	0.3
	40	82.50	89.83	90.83	3.73	4.30	4.25	4.12		
	100	84.00	93.66	92.50	3.61	3.35	2.34	5.77		

Peanut	20	106.57	109.37	109.37	3.34	5.89	1.98	1.42	0.6	0.9
	40	106.65	104.13	109.41	1.02	2.45	5.42	2.47		
	100	101.64	93.89	107.10	4.06	1.83	3.97	6.58		

Corn	20	90.10	90.98	91.72	6.77	5.63	4.89	0.89	0.6	0.8
	40	91.84	95.06	84.95	5.05	5.27	1.69	5.70		
	100	86.36	85.64	85.43	3.28	4.20	6.33	0.57		

Fish meal	20	109.85	108.88	102.31	5.02	2.96	6.18	6.33	1.2	1.5
	40	100.35	89.13	97.92	4.26	2.99	11.0	6.16		
	100	86.63	86.70	99.49	2.76	2.34	2.38	8.29		

**Table 2:** Occurrence and levels of AFB_1_ contamination in feedstuffs.

Sample matrices (n=120)	Number of samples and detected level (ng/g)						Concentration range (ng/g) (Mean±SD)
	n.d.	<4	4-20	21-50	51-100	>100	
Broken rice (30)	23	7	-	-	-	-	0.44-2.33 (0.27±0.61)

Peanut (30)	18	1	-	4	6	1	3.97-106.26 (23.29±33.76)

Corn (30)	7	10	7	5	1	-	0.88-50.29 (10.67±11.72)

Fishmeal (30)	24	4	2	-	-	-	1.06-10.35 (0.82±2.20)

## Data Availability

The data used to support the findings of this study are available from the corresponding author upon request.
